# Ocular Inflammatory Disease as a Predictor for In-Hospital Mortality in Patients Hospitalized with Disseminated Tuberculosis

**DOI:** 10.7759/cureus.956

**Published:** 2017-01-05

**Authors:** Salil Mehta

**Affiliations:** 1 Lilavati Hospital

**Keywords:** ocular, tuberculosis, mortality, disseminated

## Abstract

**Aim:**

To ascertain whether the presence of ocular inflammatory disease is a predictor for death in patients hospitalized with disseminated tuberculosis.

**Methods:**

This is an IRB-approved retrospective study of patients admitted with a diagnosis of disseminated tuberculosis within a seven-year period (2002–2009). The following data was collected from each record: age, sex, details of previous surgeries or therapy, the findings of anterior segment examination, the findings of dilated indirect ophthalmoscopy, systemic findings, investigations done, treatment rendered, and final status (died or discharged).

**Results:**

A total of 57 patients (29 males (50.8%), 28 females (49.2%) with ages ranging from 14 to 78 years (mean 41.7 years) were identified. Common presentations included fever, sepsis or neurological complaints such as headache or convulsions. Significant medical histories included acquired immune deficiency syndrome (AIDS) (n= 4), renal allograft transplantation (n=3), chronic renal failure on hemodialysis (n=3) and type 2 diabetes mellitus (n=6).

Of these, 35 patients (61.4%) had ocular tuberculosis. These included 19 males (54.2%) and 16 females (46.8%) with ages ranging from 16 to 78 years (mean 43.3 years). Current medical conditions included AIDS, renal allograft transplantation and subsequent immunosuppressive therapy, chronic renal failure on hemodialysis, and type 2 diabetes mellitus. Forty-seven of the 70 eyes (67.1%) had evidence of ocular tuberculosis. Specific presentations included 42 eyes (89.1%) with choroidal tubercles and five eyes (10.9%) with chorioretinitis. Two patients (2.8%) had disc edema. Of these 35 patients, eight (22.8%) patients died whereas 27 (77.2%) were discharged.

The remaining 22 patients (38.6%) had no ocular tuberculosis. These included 10 males (45.5%) and 12 females (54.5%) with ages ranging from 14 to 78 years (mean 39.1 years). Significant medical histories included type 2 diabetes mellitus. Of these 44 eyes, four eyes (9.09%) had non-proliferative diabetic retinopathy, two eyes (4.5%) had optic atrophy and two eyes (4.5%) had disc edema. One patient (4.5%) patient of this group of 22 died, whereas 21 (95.5%) were discharged.

We analyzed the differences in survival with Fisher’s Exact test between patients who died in the hospital and those who were discharged (statistically insignificant at p value of 0.05). Outcomes of patients with two, three, or four risk factors were analyzed using unconditional logistic regression but all tests failed to reach statistical significance.

**Conclusions:**

The presence of ocular inflammation was independent of final outcome, either singly or as part of a risk factor cluster.

## Introduction

In clinical practice, infections of the retina and choroid are commonly seen. These are usually hematogenously spread infections with a range of causative organisms including bacteria, fungi, viruses, and mycobacteria. Reported lesions in bacterial sepsis include hemorrhages, chorioretinitis, and cotton wool spots, and Bouza, et al. have suggested that the presence of these lesions may predict a higher mortality rate [1]. Mycobacterial infection may produce a spectrum of lesions including tubercles, tuberculomas, and chorioretinitis, all of which represent direct ocular tissue infection. These have been seen in up to 60% of patients with disseminated tuberculosis [2] and have a diagnostic significance as they may permit the diagnosis of systemic tuberculosis in the presence of ambiguous systemic findings [3]. However, the association between the presence of ocular tuberculosis and in-hospital mortality in patients with disseminated tuberculosis has not been studied so far. We conducted a retrospective study to address this issue. Lilavati Hospital and Research Centre issued IRB approval for this study. Informed consent was obtained from the patients for this study.

## Materials and methods

We conducted an IRB-approved retrospective study. We analyzed the records of patients who were admitted with disseminated tuberculosis in the intensive care unit or wards of a tertiary level hospital within a seven-year period (2002–2009). The following were excluded from further analysis: patients whose records were unavailable and those who were discharged against medical advice.

The records were studied for baseline demographic data (age and sex), and details of previous surgeries or antibiotic therapy prior to admission were ascertained. Ocular data included the findings of anterior segment examination (if available) and the findings of dilated indirect ophthalmoscopy. Systemic findings recorded included the location and severity of tubercular disease and an evaluation for HIV 1/ 2, and diabetes mellitus. Laboratory investigations and radiological tests (plain X-rays, computed tomography (CT) scans, and ultrasound evaluations) were recorded. Relevant microbiological evaluation of sputum, tissues or histopathology of these tissue samples were recorded. The treatment rendered and final status (died or discharged) were noted.

This diagnosis of disseminated tuberculosis was confirmed in each case by radiological and/or clinical evidence of tuberculosis in at least two distinct organ systems. The clinical pattern of tuberculosis was reviewed to permit a diagnosis of a miliary or non-miliary pattern of disseminated tuberculosis. “Miliary” was taken to mean a classical radiological appearance of extensive reticular-nodular pattern of lung shadowing seen on chest radiography.

We hypothesized four risk factors: 1) age greater than 50 years, 2) the presence of pre-existing immunosuppression, 3) the presence of diabetes mellitus, and 4) the presence of ocular tuberculosis. We constructed an Excel worksheet (Microsoft Corp, WA, USA) with this data. Further statistical analysis was done with Epi Info™ (CDC, GA, USA). Initially, we analyzed the differences in survival with Fisher’s Exact test between patients who died in the hospital and those who were discharged. Corrections were applied using an unconditional logistic regression, adjusting for the presence of immunosuppression as a confounding factor, to assess whether the presence of ocular tuberculosis alone was a predictor of death. Next we created three categories of patients—those with two, three, or four risk factors—and analyzed using unconditional logistic regression whether these “risk factor clusters” could serve as predictors of in-hospital mortality in these patients.

## Results

A total of 57 patients (29 males (50.8%), 28 females (49.2%) with ages ranging from 14 to 78 years (mean 41.7 years) were identified. Common presentations included fever, sepsis or neurological complaints such as headache or convulsions. Significant medical histories included acquired immune deficiency syndrome (AIDS) (n=4), renal allograft transplantation (n=3), chronic renal failure on hemodialysis (n=3), and type 2 diabetes mellitus (n=6).

Of these, 35 patients (61.4%) had ocular tuberculosis. These included 19 males (54.2%) and 16 females (46.8%) with ages ranging from 16 to 78 years (mean 43.3 years). Current medical conditions included AIDS (n=4, 11.4%), renal allograft transplantation and subsequent immunosuppressive therapy (n=3, 8.5%), chronic renal failure on hemodialysis (n=3, 8.5%) and type 2 diabetes mellitus (n=3, 8.5%). Forty-seven of the 70 eyes (67.1%) had evidence of ocular tuberculosis. Specific presentations included 42 eyes (89.1%) with choroidal tubercles and five eyes (10.9%) with chorioretinitis. Two patients (2.8%) had disc edema. Of these 35 patients, eight (22.8%) patients died whereas 27 (77.2%) were discharged.

The remaining 22 patients (38.6%) had no ocular tuberculosis. These included 10 males (45.5%) and 12 females (54.5%) with ages ranging from 14 to 78 years (mean 39.1 years). Significant medical histories included type 2 diabetes mellitus (n=3, 13.6%). Of these 44 eyes, four eyes (9.09%) had non-proliferative diabetic retinopathy, two eyes (4.5%) had optic atrophy and two eyes (4.5%) had disc edema. One patient (4.5%) patient of this group of 22 died, whereas 21 (95.5%) were discharged (Tables 1-2).

**Table 1:** Showing the correlation between the presence of ocular tuberculosis and in-hospital mortality (Fisher's Exact test is 0.052)

Ocular Tuberculosis	0	1	Total
0	22	1	23
Row%	95.7	4.3	100
Col%	45.8	11.1	40.4
1	26	8	34
Row%	76.5	23.5	100
Col%	54.2	88.9	59.6
Total	48	9	57
Row%	84.2	15.8	100
Col%	100	100	100

**Table 2:** Unconditional logistic regression showing the contribution of various factors to the outcome. (DM = diabetes mellitus; IS = immunosuppression; OTB = ocular tuberculosis)

Term	Odds Ratio	95%	C.I.	Coefficient	S.E.	Z-statistic	P-value
Age Gr > 50 yrs	0.36	0.03	3.72	-1.01	1.18	-0.85	0.39
DM	23.00	0.81	651.3	3.13	1.70	1.83	0.06
IS	0.46	0.08	2.59	-0.76	0.87	-0.87	0.38
OTB	7.92	0.65	97.11	2.07	1.27	1.62	0.14
Sex	1.45	0.22	8.32	0.37	0.88	0.42	0.67
Constant	*	*	*	-1.93	1.91	-1.00	0.31

Although eight of nine (88%) who died had ocular tuberculosis, there were 26 of 34 (77%) who had ocular tuberculosis but were discharged. A logistic regression to correct for immunosuppression showed the contribution of various factors to the outcome. Diabetes mellitus and ocular tuberculosis had the largest coefficients (3.13 and 2.07) but both failed to reach significance at p<0.05 (Tables 3-5).

**Table 3:** Unconditional logistic regression with patients having two risk factors correlated with the outcome (ISF = immunosuppression factors; OTB = ocular tuberculosis)

Term	Odds Ratio	95%	C.I.	Coefficient	S.E.	Z-statistic	P-value
ISF>= 2	1.31	0.26	6.43	0.27	0.81	0.33	0.73
OTB	7.30	0.81	65.87	1.98	1.12	1.77	0.07
Constant	*	*	*	-3.33	1.25	-2.65	0.007

**Table 4:** Unconditional logistic regression with patients having three risk factors correlated with the outcome (ISF = immunosuppression factors; OTB = ocular tuberculosis)

Term	Odds Ratio	95%	C.I.	Coefficient	S.E.	Z-statistic	P-value
ISF>= 3	0.25	0.02	2.15	-1.38	1.09	-1.26	0.20
OTB	5.50	0.61	49.3	1.70	1.11	1.52	0.12
Constant	*	*	*	-1.70	1.50	-1.13	0.25

**Table 5:** Unconditional logistic regression with patients having four risk factors correlated with the outcome (ISF = immunosuppression factors; OTB = ocular tuberculosis)

Term	Odds Ratio	95%	C.I.	Coefficient	S.E.	Z-statistic	P-value
ISF>= 4	1.09	0.38	3.09	0.09	0.52	0.17	0.85
OTB	6.11	0.54	69.13	1.81	1.23	1.46	0.14
Constant	*	*	*	-3.13	1.05	-2.97	0.002

A logistic regression analysis for patients with two, three, or four risk factors revealed no statistically significant p values for a relationship between these risk factor clusters and death.

## Discussion

We studied 57 adult hospitalized patients of disseminated tuberculosis. All these patients had multiple foci of tubercular infection and covered the entire spectrum of clinical disease from mild fever to septic shock. A majority of these patients had ocular tuberculosis confirming a high prevalence in these patients. The correlation between the presence of ocular tuberculosis and in-hospital mortality in these patients has not been reported so far.

In patients with bacterial sepsis, a large body of literature has reported the outcome and predictors of mortality in these patients. Most studies have suggested the use of some form of multifactorial score to grade these patients and study the final outcome. Ter Avest, et al. have suggested that a higher abbreviated Mortality in Emergency Department Sepsis (MEDS) score, a lower hemoglobin or significant beta-blocker use was associated with an increased mortality [4]. Vorwerk, et al. also noted an association between the MEDS score, the Modified Early Warning (MEW) score and near-patient-test (NPT) lactate levels in predicting early mortality [5].

The overall mortality and its predictive factors in patients with disseminated tuberculosis has been less comprehensively reported. In a study of 109 adults, Maartens reported 26 deaths (24%). Using logistic regression, they identified age (above 60 years), lymphopenia, thrombocytopenia, hypoalbuminemia, elevated transaminase levels, and treatment delay as important predictors of mortality [6]. Kim, et al. showed a mortality of 21% in their series of 38 non-HIV (non human immunodeficiency virus) infected patients and identified female sex and altered mental status as risk factors [7]. Interestingly, in a study of 100 non-HIV infected adults from India, Sharma, et al. reported a mortality of 12% and showed that hypoalbuminemia, hyponatremia and presence of crepitations on auscultation were independent predictors of mortality [8]. No ophthalmological data was reported in any of these studies.

The strength of our study is the relatively large size of our cohort. Weaknesses include its retrospective nature, our inability to conduct a follow-up (including readmissions) beyond the current hospitalization, and a sample size that did not allow further subgroup studies based on demographics or the grade of sepsis. Further, the majority of patients were in a critical condition and ocular evaluation beyond dilated indirect ophthalmoscopy was not possible. Thus the presence and significance of anterior segment inflammation could not be evaluated.

In this study, the presence of ocular inflammation alone or as part of the risk factor cluster we hypothesized was independent of the final outcome. This may be due to the fact that mortality in patients of disseminated tuberculosis may have several contributing factors including the sites of infection, time to detection, drug resistance patterns, given treatment, and possibly pre-existing diseases. A score that includes ocular findings and systemic/metabolic findings at admission may need to be developed.

## Conclusions

Patients of suspect disseminated tuberculosis should continue to be examined for diagnostic reasons, but the presence of ocular tuberculosis is not a predictor of in-hospital mortality either singly or as part of this risk factor cluster.
